# Opportunities for machine learning to predict cross-neutralization in FMDV serotype O

**DOI:** 10.1371/journal.pcbi.1013491

**Published:** 2025-09-17

**Authors:** Dennis N. Makau, Jonathan Arzt, Kimberly VanderWaal

**Affiliations:** 1 Department of Biomedical and Diagnostic Sciences, College of Veterinary Medicine, University of Tennessee, Knoxville, Tennessee, United States of America; 2 Department of Veterinary Population Medicine, College of Veterinary Medicine, University of Minnesota, Minnesota, United States of America; 3 Department of Public Health, Pharmacology and Toxicology, Faculty of Veterinary Medicine, University of Nairobi, Kenya,; 4 Plum Island Animal Disease Center, Agricultural Research Service, U.S. Department of Agriculture, Greenport, New York, United States of America; Pennsylvania State University, UNITED STATES OF AMERICA

## Abstract

Accurately estimating cross-neutralization between serotype O foot-and-mouth disease viruses (FMDVs) is critical for guiding vaccine selection and disease management. In this study, we developed a machine learning approach to estimate r1 values—an established measure of antigenic similarity—using VP1 sequence data and published virus neutralization titer (VNT) results. Our dataset comprised 108 serum-virus pairs representing 73 distinct FMDV strains. We applied Boruta feature selection and random forest classifiers, optimizing model performance through tenfold cross-validation and sub-sampling to address class imbalance. Predictors included pairwise amino acid distances, site-specific polymorphisms, and differences in potential N-glycosylation sites. Using a 0.3 r1 threshold to define cross-neutralization, the final model achieved high accuracy (0.96), sensitivity (0.93), and specificity (0.96) in training, and performed robustly on independent test sets - accuracy was 0.75 (95% CI 0.60 and 0.90), F1 score 0.86% and PPV 0.77. Importantly, key VP1 residues—positions 48, 100, 135, 150, and 151—emerged as strong predictors of antigenic relationships. Our results demonstrate the utility of integrating routinely generated genomic data with machine learning to inform vaccine candidate selection and anticipate immune interactions among circulating FMDV strains. This approach offers a practical tool for accelerating vaccine decision-making and can be adapted to other FMDV serotypes. The latest version of the r_1_ predictive model is available for access via a Shiny dashboard (https://dmakau.shinyapps.io/PredImmune-FMD/).

## Author summary

Foot-and-mouth disease (FMD) is a highly contagious virus that threatens the health of livestock and the livelihoods of farmers around the world. Controlling outbreaks depends on using vaccines that protect against the specific virus strains circulating in the field. However, testing whether a vaccine will be effective typically requires complex laboratory experiments that are not always possible during urgent outbreaks. In this study, we developed a computer-based method using machine learning to predict how closely a vaccine strain matches a field virus by comparing their genetic sequences, focusing on a key virus protein involved in immunity. We collected data from 108 tests reported in previous studies and trained a model that accurately—over 90% of the time—identified vaccine and virus strain combinations likely to provide good protection. This approach can speed up vaccine selection, help prepare for outbreaks faster, and reduce reliance on costly lab tests, particularly in regions with limited resources. Importantly, we have also translated our model into an interactive, user-friendly tool called PredImmune-FMD available at https://dmakau.shinyapps.io/PredImmune-FMD/, enabling researchers, field veterinarians, and decision-makers to explore predictions in real time.

## Introduction

Foot and mouth disease (FMD) is a viral disease caused by the FMD virus (FMDV), a member of the *Piconaviridae* family [1] that affects cloven-hoofed ungulates. Though typically not fatal, the impact of FMD on food security and livelihoods in endemic countries (particularly low-and middle-income countries (LMICs)) cannot be overemphasized [2]. FMD also continues to be a major stumbling block to livestock production and global trade in many parts of the world, especially with the continued threat of emergence and introduction of new FMDV lineages/strains into FMD-free countries. As such, numerous efforts and pathways to achieve and maintain disease free status in most countries has required, among other things, the ability and capacity to identify viruses and vaccinate animals with appropriate vaccines for countries where elimination is yet to be achieved. Although there are 7 documented antigenically distinct serotypes (O, A, C, SAT1, SAT2, SAT3 and Asia1), serotype A and O have been reported to be the most common causes of FMD globally [3–6].

Continued efforts for disease management in FMD include identifying suitable vaccine candidates and formulating vaccines for at-risk animal populations. To identify these candidates, a process known as vaccine matching is conducted, using viral neutralization titer (VNT) assays to assess the cross-reaction and cross-neutralization potential of candidate vaccines against field strains. The effectiveness of cross-neutralization is expressed as the r_1_ value, which is defined as the ratio of the reciprocal heterologous serum titer (typically of a field strain) relative to the reciprocal homologous titer (usually of the vaccine strain or vaccine candidate). An r_1_ value ≥ 0.3 suggests potential cross-protection between the virus strains, whereas values < 0.3 indicate a lack of cross-protection [7,8].

Generating r_1_ data for comparison demands significant effort, precision, and laboratory resources. This involves producing anti-sera in live animals and running *in vitro* assays to evaluate the cross-reactivity of target viruses with various sera samples. Advances in computational biology empower us to harness machine learning and artificial intelligence to build computation tools aimed at estimating the cross-neutralization potential between different viruses. While previous studies have explored predictive models of FMDV antigenicity, they have varied objectives. Some focused on predicting FMDV antigenicity and identifying antigenicity descriptors [9], identifying and predicting specific epitopes and genomic regions of antigenic importance [10,11], or developing intricate models to forecast antigenic changes among SAT 1 and 2 FMDV serotypes [12–14]. There is hardly any literature specific to serotype O despite it being one of two widely occurring serotypes, nor has research on the application of machine learning to estimate r_1_ values been published. Although there have been some divergent views about the adequacy of using r_1_ values in identifying potential cross-protection [7], it is still one of the metrics relied upon when interpreting data generated from *in vitro* VNT assays and selecting candidate vaccine strains. Successful application of machine learning to aid in decision making when selecting vaccine candidates and immunization programs has been demonstrated in diseases such as influenza and dengue virus [15–17]. Our intention in this study was to develop a simple, yet robust, predictive tool that can be used to estimate potential cross-neutralization between viruses by leveraging machine learning algorithms and genetic characteristics of FMDV.

Therefore, the objective of this study was to develop an algorithm to estimate potential cross-neutralization between strains of serotype O FMDV using r_1_ values and identify important genomic sites that influence high or low r_1_ values between viruses. The ability to distinguish and estimate the potential for cross-protection between different cocirculating FMD viruses *in silico* will support decision-making by vaccine developers in vaccine candidate matching, animal health agencies/institutions in decision-making for herd immunization practices, and overall disease preparedness and response in cases of emergent strains especially for serotype O.

## Results

### Descriptive analysis

In this dataset, assembled from peer-reviewed manuscripts reporting cross-neutralization assays conducted consistent with World Organization for Animal Health (WOAH) guidelines, there were 108 observations (serum-virus pairs) from 73 distinct viruses for which r_1_ values had been reported. In summary, the mean r_1_ value was 0.22 ± 0.23 (SD), with a range of (0.0-0.94) and a median of 0.16. Upon dichotomizing the data using the 0.3 threshold as cutoff, the two resulting groups used in subsequent modelling comprised of 87 pairs in the non-cross-neutralizing group (< 0.3 r_1_ values) and 21 in the cross-neutralizing group (≥ 0.3 r_1_ values).

**Fig 1:**
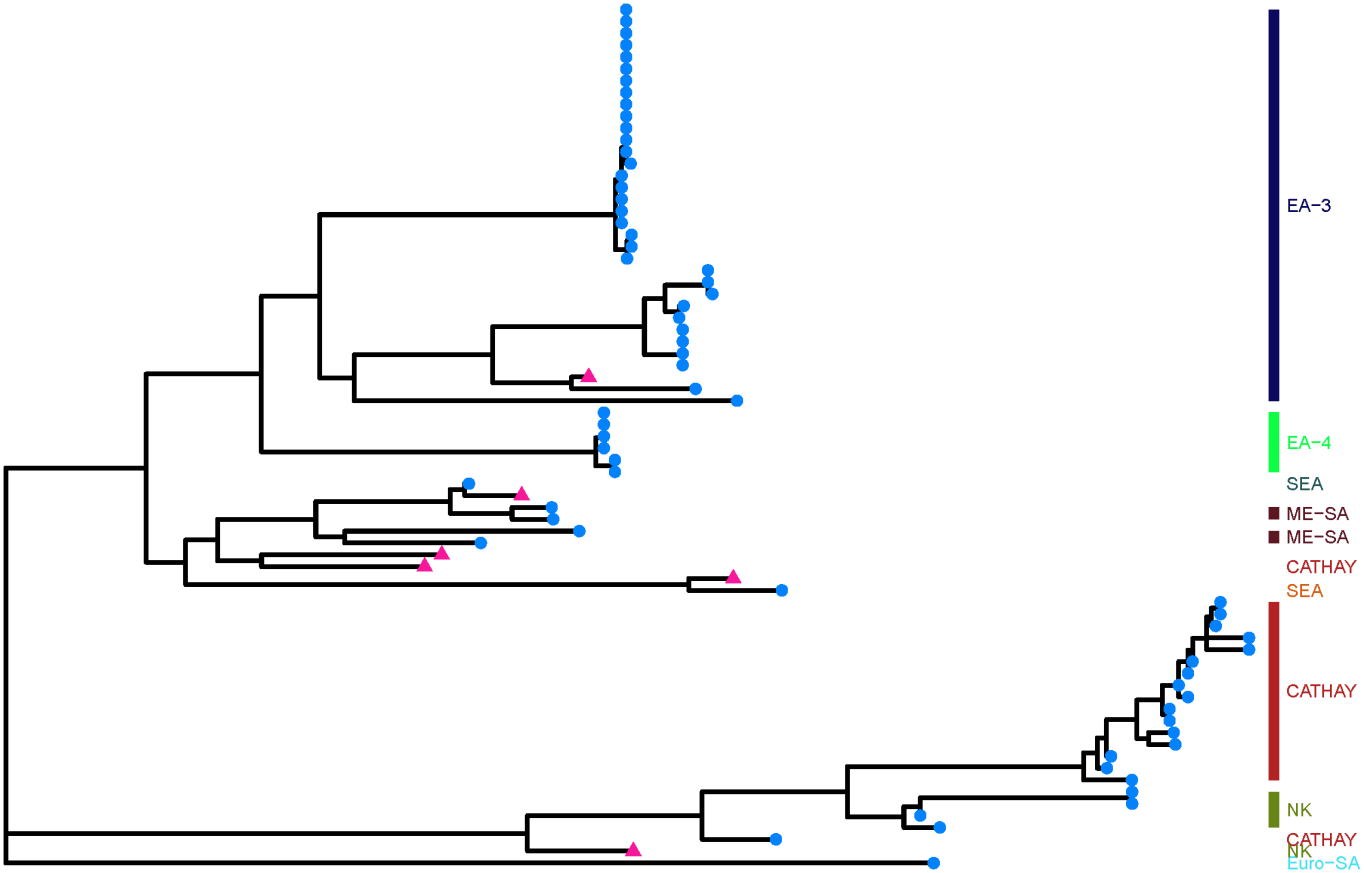
Maximum likelihood tree depicting the phylogenetic distribution of 73 foot and mouth viruses (reference serum (pink triangles) and field strains (blue circles)) representing serotype O topotypes used in the development of an r_1_ predictive model. The colored bars on the right of the tree represent the different topotypes included in the data (*NK = Not Known*).

**Fig 2:**
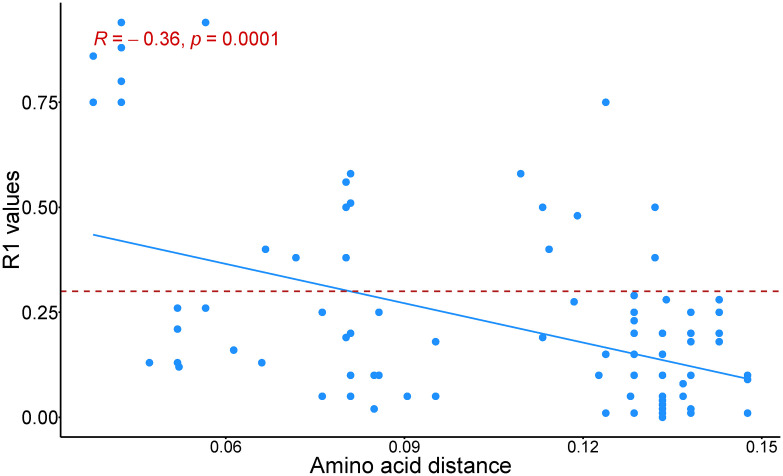
Correlation plot depicting an inverse moderate Spearman’s correlation (blue) between pairwise amino acid distance of the VP1 region for serotype O foot and mouth disease viruses and r_1_ values obtained from *in vitro* virus neutralization assays. The red-dotted line represents the cut-off of 0.3 threshold for potentials cross-neutralization between serum-virus pairs.

Additionally, upon phylogenetic assessment, our dataset included viruses belonging to five topotypes for FMDV serotype O and the serum/vaccines used in the assays were evenly distributed among the topotype groupings (Fig 1). The mean VP1 pairwise amino acid distance for the pairs was 0.12 ± 0.03 with a range of 0.04 – 0.15 and median of 0.13. There was a significant (p = 0.0001) moderate negative correlation (Spearman rho = -0.4) between pairwise amino acid distance and r_1_ values between the pairs (Fig 2). While a moderate negative correlation was expected, the relationship is relatively weak likely because not all amino acid changes are antigenically meaningful—only substitutions at or near immunodominant or surface-exposed regions tend to influence neutralization, and other biological factors such as host immune variability or compensatory mutations may also play a role.

**Fig 3:**
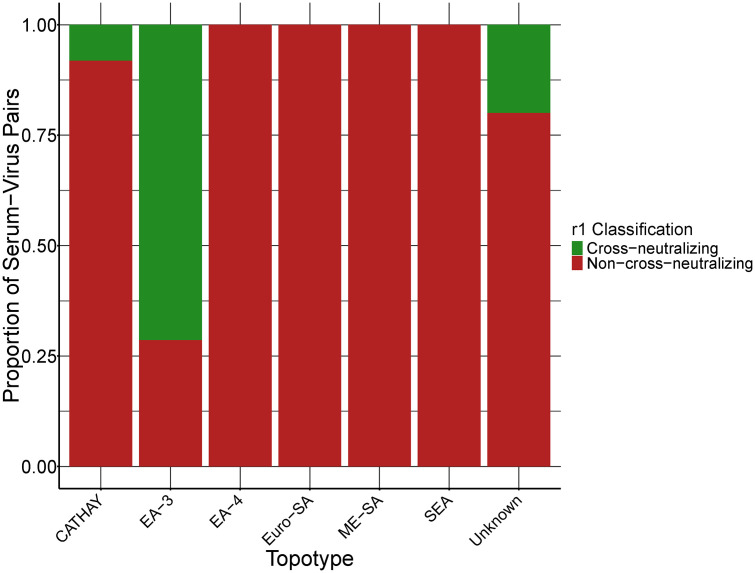
Observed cross-neutralization patterns by FMDV topotype among 108 serum-virus pairs. Bars represent the proportion of serum-virus pairs classified as cross-neutralizing (r1 ≥ 0.3; green) or non-cross-neutralizing (r1 < 0.3; red), based on laboratory-generated r1 values. Topotype assignments are based on the field virus only. Three virus strains lacked topotype information and are labeled as “Unknown”.

To explore potential topotype-level patterns in antigenic compatibility, we summarized the observed laboratory r1 classifications across viral topotypes. Of the 108 serum-virus pairs used in model development, 70 had known topotype annotations for the field virus. As shown in Fig 3, cross-neutralizing outcomes (r1 ≥ 0.3) were more common among some topotypes—such as EA-3 and SEA—while others, including CATHAY and ME-SA, were predominantly non-cross-neutralizing. These results suggest that cross-neutralization patterns may partly reflect underlying antigenic variation between topotypes. However, interpretation should be cautious given the limited and uneven distribution of samples across topotypes.

Based on the phylogenetic tree (Fig 1) and correlation plot (Fig 2), viruses with greater VP1 sequence similarity generally had higher r1 values. Many of the cross-neutralizing pairs fell within the EA-3 and SEA topotypes, which also had a higher proportion of r1 ≥ 0.3 cases (Fig 3). So, genetic clustering within topotypes often points to antigenic similarity. That said, there are some notable outliers—phylogeny does not always tell the whole story. It is likely that specific amino acid changes at key antigenic sites modulate broader genetic relationships.

### Random forest model performance and predictions

The best performing model had an accuracy of 0.96 (95% CI 0.88-0.99), F1 score of 0.899, sensitivity and specificity of 0.93 and 0.96, and negative and positive predictive values of 0.98 and 0.87, respectively, on training data [n = 70: 0 (non-cross-reacting) =56, 1 (cross-reacting) =14]. On one testing dataset (n = 17: 0 = 14,1 = 3), the model accuracy was 0.88 (95% CI 0.64-0.99), F1 score of 0.75, sensitivity of 1.00 and specificity of 0.86, and PPV and NPV of 0.60 and 1.00, respectively. The model performance on the second test/validation data (n = 21: 0 = 17, 1 = 4) was 1.00 accuracy (95% CI 0.84-1.00), F1 score of 1.00, sensitivity and specificity of 1.00 and 1.00, and PPV and NPV of 1.00 and 1.00, respectively. When applied to external data published by other studies (n = 31, 0 = 7, 1 = 24) [18–21], the model accuracy was 0.75 (95% CI 0.60 and 0.90), sensitivity/recall of 0.96 and F1 score 0.86% and PPV 0.77 (S1 Table).We also mapped the validation dataset to the original data to visualize the distribution of topotypes in the validation dataset. All topotypes in the training dataset were represented and proportionally distributed in the tree topology (S1 Fig).

**Fig 4:**
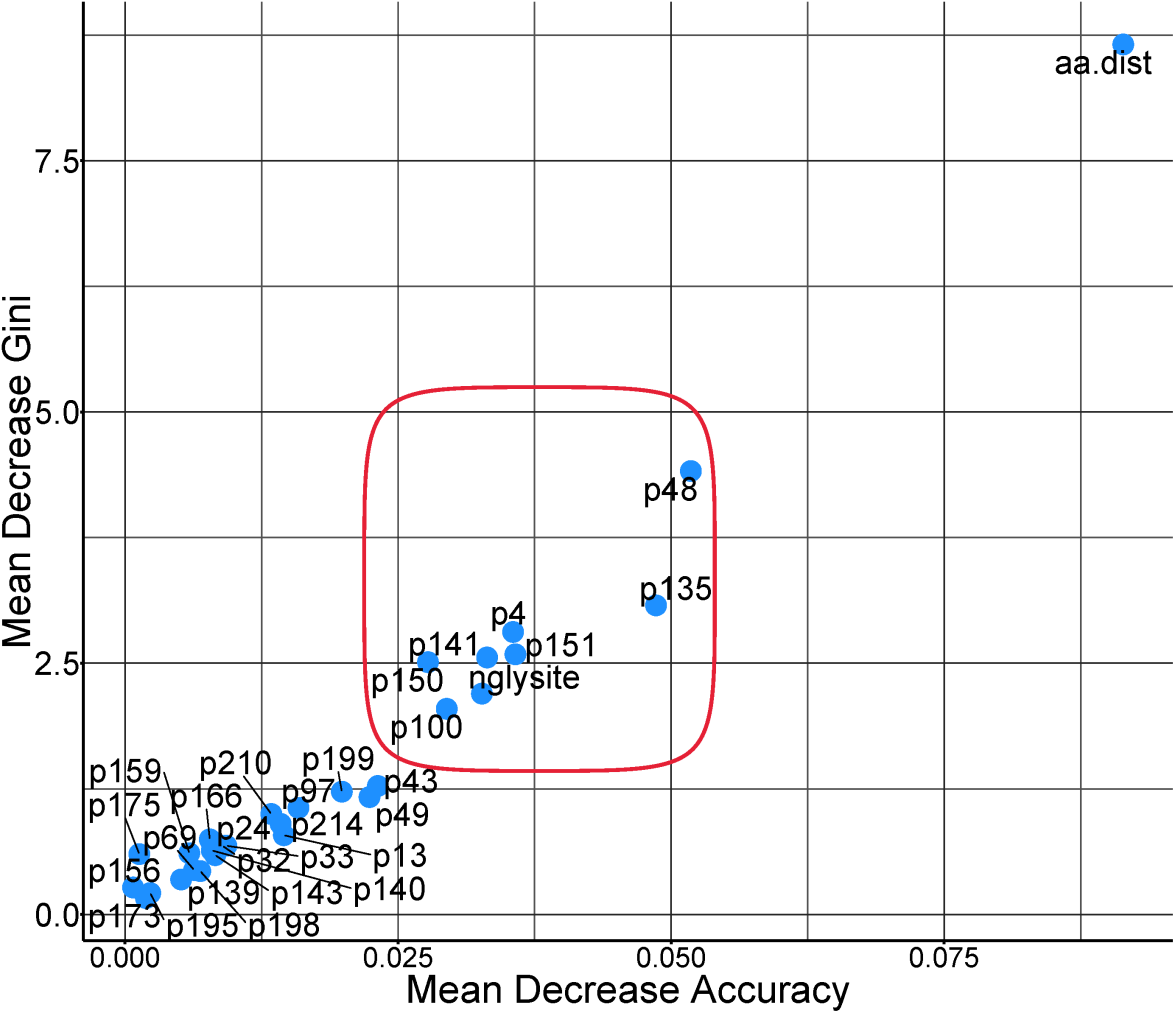
Multiway importance plot for model covariates included in the final random forest model predicting r_1_ values in VP1 region for serotype O foot and mouth disease virus. Feature labels in the graph indicate the specific amino acid site (p) while ‘aa.dist’ indicates the pairwise amino acid distance between serum-virus pairs, ‘nglysite’ similarity or difference in positions for potential N-glycosylation.

From the variable importance analysis that extracted which predictors had the most influence in the models’ predictive ability and accuracy, several sites were ranked highly besides the pairwise distances between amino acid sequence. These include amino acid positions 48, 100, 135, 150, and 151 in the VP1 region (Fig 4).

To evaluate the structural impact of SMOTE, we performed principal component analysis (PCA) on the training data before and after augmentation (S2 Fig). The resulting PCA plots revealed that SMOTE-generated samples for the minority class (cross-neutralizing) and expanded the occupied feature space without introducing extreme outliers. Importantly, synthetic points remained within the overall range of the original data and appeared biologically plausible. The first two principal components explained over 35% of total variance in both datasets, and class separation slightly improved following SMOTE.

## Discussion

The suitability of using *in silico* methods to estimate *in vivo* cross-protection in FMD [22] and r_1_ values as opposed to other measures of immune response and cross-reactivity has been argued by several studies [7,22–25]. Although this model is based on r_1_ values, the approach can be modified to accommodate other measures of antigenic variability, like raw VNT results. Since r_1_ values are still the most-used option when conducting vaccine matching and selection of candidates, whether achieved through simple ELISA or a modified combination of techniques to estimate cross-protection, a model like ours could aid in the identification of potential cross-neutralizing and non-cross-neutralizing sera. This streamlines the selection of potential vaccine candidates and facilitates immediate comparisons with field strains where necessary. Moreover, when applied in non-research settings, animal health agencies/institutions can easily input sequences to the model from isolates or clinical samples and promptly identify potential options for immunization from commercially available vaccines. The continued improvement and accessibility of sequencing technology such as Minion Nanopore sequencing, which can be deployed in LMIC settings, may also increase the frequency and capacity of in-country generation of sequences (especially in countries where access to reference labs is limited), complementing the utility of predictive tools supported by this model. That said, predicted outputs are only estimates, and should be confirmed experimentally.

We developed a random forest classification model that estimates potential cross-neutralization between serum and viruses (i.e., r_1_ values) for viruses belonging to serotype O FMDV with an accuracy of more than 85% (up to 94%) and F1 score of 86%. This model adds to a growing body of research leveraging bioinformatic data to streamline and enhance our understanding on viral antigenicity and host immune response as exemplified in diseases like influenza [16,17,26–29]. Optimized and robust predictive models can be valuable in decision making and implementing strategic interventions towards the control of FMDV. The performance of this model so far is encouraging, and could be further improved with inclusion of antigenically diverse viruses, such as those in Bachanek-Bankowska [18]. However, even with improved predictive ability to accurately classify cross-neutralizing serum-virus pairs, there are drawbacks to *in silico* modelling for biological processes. The immune response is a composite process which involves the interplay of multiple compartments of the host immune response and different factors (e.g., environment and host genetics) that may influence host-virus interactions. Such factors contribute to why measures of cross-reactivity *in vitro* often do not translate well to *in vivo* cross-protection in the field [30–33].

**Fig 5:**
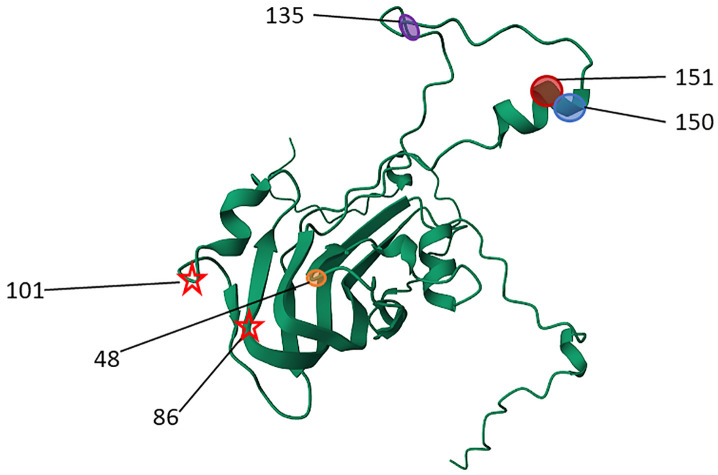
3D representation of the VP1 region of foot and mouth disease virus serotype O. Amino acid sites depicted with colored oval shapes were among the top amino acid sites identified as influential to model accuracy. The red stars indicate positions predicted to most frequently undergo N-glycosylation. The residues labeled 135, 150, and 151 are part of the surface-exposed GH loop a major target for neutralizing antibodies. Residues labeled 48, 86, and 101 are likely part of the beta-sheet core of the VP1 protein and are less likely to be surface-exposed.

By highlighting the highly ranked/important amino acids in the VP1 region, we posit that those amino acids may potentially play an important role in host immune induction and response as differences in those sites appear to be important for estimating the r_1_ values between serum-virus pairs. Other studies [11,13] also support the existence of immunodominant sites in the VP1 region. Further research may be beneficial to better understand how selection pressures at these sites may contribute to challenges with vaccine effectiveness, causes of vaccine failure, breakthrough outbreaks in vaccinated populations, and strain emergence [30]. In these data, seven amino acid positions were most influential in the ability of our model to accurately distinguish between cross-neutralizing (≥0.3) vs non-cross-neutralizing (<0.3). These were amino acids in positions 4, 48, 100, 135, 141, 150 and 151. Except position 4 and 100, all these amino acids are part of immunogenically important regions described as the B-C and G-H loops in the VP1 protein of serotype O [6] in which certain mutations in combination or independently have been thought to confer serological heterogeneity among FMDVs [34]. In sequences used for this study, N-glycosylation was predicted to occur most often at sites 86, then 101, and least often at site 132. From estimated 3D projection of the VP1 region [35], sites 86 and 101 were in regions predicted to be part of the core while 132 was part of the GH loop (surface proteins/exposed), suggesting their potential to participate in host immune interactions. Fig 5 depicts a 3D representation of these amino acid sites [36].

To expand on their potential roles in host immune induction, residues 135, 150, and 151—all within the G-H loop—stand out as part of a surface-exposed, immunodominant region that consistently elicits strong neutralizing antibody responses in FMDV [37]. Residue 141, located nearby, may also influence the structural integrity of this epitope and affect antibody accessibility. In contrast, positions 48 and 100 are more likely to contribute indirectly by stabilizing the conformation of immunogenic loops or influencing overall capsid architecture. The consistent appearance of these sites among top-ranking model features highlights their potential immunological relevance. However, experimental validation through techniques such as site-directed mutagenesis and epitope mapping remains essential to confirm their contributions to cross-neutralization and vaccine performance.

Glycosylation at or near antigenic sites may modulate epitope accessibility, potentially influencing cross-reactivity by shielding neutralizing regions or altering antibody binding dynamics. While this structural mapping offers preliminary biological interpretation, statistical importance alone does not confirm immunological relevance. Future studies that integrate site-directed mutagenesis or epitope mapping will be essential to validate the functional roles of these residues in mediating cross-neutralization.

The wide confidence interval (95% CI 0.64–0.99) suggests that some misclassification occurred. This uncertainty may reflect not only model limitations but also inherent variability in how r1 values are derived across studies. While we selected studies using standardized WOAH protocols, differences in laboratory procedures, reagents, or interpretation can still introduce variability. Our model does not account for all these factors and others that may influence host-pathogen interactions. Nevertheless, using available data, we were able to accurately categorize 8 or 9 of every 10 serum-virus pairs for serotype O as cross-neutralizing or non-cross-neutralizing (based on r_1_ values). When applied to a completely external dataset, the model performed comparably, accurately predicting 7 out of 10 serum-virus pairs. We attempted to mitigate these limitations analytically by including lab as model covariate in the model but it was not among highly ranked predictors in the model outputs. Future studies could consider building models based on raw VNT assay data and metrics such as IgG1/IgG2 subclass ratios, IgG avidity and vaccine potency [7,38]. Further research and generation of such data would be a valuable addition to this topic and likely improve utility of these models.

As such, continued improvement of *in silico* models is necessary both with diverse datasets and potential validation with *in vivo*/field application data as attempted in this study. That limitation notwithstanding, with the limited data used here, the model can at least aid in filtering potential candidates to be considered for in-depth *in vitro*/*in vivo* assays, which would save resources and increase efficiency of the decision-making process and FMD management. Additionally, in future work, incorporating weighted modeling strategies that reflect evolutionary or antigenic relationships among viral strains could provide additional insight and improve prediction of cross-protection in diverse settings.

Lastly, VPs 2 and 3 also likely play a role in host-virus interaction and immune response. Mutations in these regions may influence antigenic variability and neutralization outcomes. However, since we only relied on publicly available secondary data, we did not have access to full genome sequences for all 73 viruses included in the study necessitating restricting our analysis to VP1. While VP1 remains the most commonly sequenced and well-characterized region, broader genomic coverage—across both structural and non-structural proteins—could improve model performance. Future efforts will explore the benefits of a broader genomic analysis and more sensitive threshold of cross-protection.

## Conclusion

This study adds to the growing body of literature that machine learning can be applied to genetic/genomic data to achieve a more nuanced analysis of the relationship between genetic variability and cross-recognition of viruses by host immune systems. Such models can support immunization as a pathway to disease management. In this study, we demonstrate the opportunities and potential for leveraging routinely generated sequence data and the application of a machine learning model to streamline the process of decision-making in vaccine development and application to control FMD, especially serotype O (but also applicable to other serotypes). Specifically, by obtaining an accurate model with high sensitivity and specificity, appropriate vaccine candidates may be able to be selected more quickly, although *in vivo* experiments would still ultimately be necessary to assess cross-protection. Also, this capability would enable tailoring immunization protocols for use in the field with a faster turnaround time for decision-making. Lastly, outputs from such a model can be combined with other mathematical models to understand drivers and trends of viral emergence, especially the role of immune pressure in driving the evolution and spread of FMDV. The latest version of the r_1_ predictive model is available for access via a Shiny dashboard (https://dmakau.shinyapps.io/PredImmune-FMD/).

## Materials and methods

### Data preparation

We obtained data from published manuscripts on r_1_ values for FMDV serotype O. Viruses utilized in these papers came from 14 countries. Relevant manuscripts were identified through targeted searches in Google Scholar and PubMed using terms such as *“FMDV and r1 values”* and *“FMDV and vaccine matching”*. This yielded four eligible studies, summarized in Table 1 [39–42].

**Table 1:** Summary of studies reporting r_1_ values for FMDV serotype O included in this analysis. Data were extracted from four published manuscripts identified through targeted searches in Google Scholar and PubMed using terms such as “FMDV and r1 values” and “FMDV and vaccine matching.” The compiled dataset spans 108 virus–serum pairs from 14 countries and includes diverse topotypes within serotype O.

Study	# of r1 pairs	Topotypes Represented
Tesfaye et al. 2020	39	EA-3, EA-4
Yang et al. 2014	30	ME-SA, EA-3, SEA, etc.
Upadhyaya et al. 2021	35	PanAsia-2, IND-R2/75
Singanallur et al. 2022	4	ME-SA

To mitigate limitations associated with dataset imbalance and methodological variability across studies [7,43], we adopted two strategies. We employed strict selection criteria for the inclusion of manuscripts. Specifically, studies were only included if they: provided r1 values derived from standardized two-dimensional virus neutralization tests (2D-VNT), used bovine vaccinate sera, and explicitly followed or were consistent with World Organization for Animal Health (WOAH) guidelines [44]. For each included study, we reviewed the methods section to verify that their virus neutralization protocols either directly cited the WOAH Manual or described procedures consistent with it—specifically, two-dimensional VNTs using bovine vaccinate sera and the r1 ratio calculated as the heterologous-to-homologous neutralization titer.

Some of these studies utilized antisera generated from vaccines or vaccine candidates (some of which were never commercialized); in this paper, we refer to both as “reference” strains. A full list of GenBank accession numbers for field and reference strains, along with their source publications, is provided in S1 Table and hosted at Zenodo [https://doi.org/10.5281/zenodo.15923491]. The r_1_ values indicate the serological compatibility between the reference strain/candidate and the field isolates, determined by comparing the reactivity of the heterologous versus homologous virus to the antisera produced against the specific viruses. The ratio of the heterologous to homologous neutralization titers (expressed as reciprocals) between field isolates and reference antisera serve as a measure of cross-neutralization.

Since the majority of the published studies only had VP1 sequence data publicly available on GenBank, we downloaded sequence data for the VP1 gene of 73 distinct viruses used in the cross-reactivity and generation of r_1_ data summarized in the manuscripts described above; any whole genomes available were trimmed to the VP1 region using Aliview 1.26 [45]. Accession numbers to the sequences used in this analysis are available in supplemental material 1. Subsequent steps in data preparation included: *Alignment –* Using MUSCLE in Aliview 1.26 [45], we aligned and translated sequences into amino acid sequences. *Distance calculation and coding –* Using MEGA X [46], we calculated Poisson corrected amino acid distance between all pairs [46,47]. Then using R packages *stringr* [48], *bioseq* [49], *ape* [50] and *tidysq* [51], we coded differences at amino acid sites using R v4.2 software [52] assigning ‘0’ if they shared the same amino acid at that site, and ‘1’ if they differed. *Glycosylation site analysis* – We scanned the VP1 region for potential N-glycosylation sites [53,54], and coded pairs with identical sets of inferred glycosylated sites as ‘0’, and non-identical sets as ‘1’*. Data compilation and model features:* We concatenated the following data into a single data frame for modeling: r1 values as reported in the manuscripts, pairwise amino acid distances calculated in MEGA X, site-wise differences in amino acid sequences, glycosylation site differences. The resulting data frame comprised 108 observations (serum-virus pairs). Although some studies on influenza have explored the pros and cons of weighting of specific amino acid sites based on *a priori* knowledge of their evolutionary history and influence on antigenicity [55,56], our modeling approach was naïve to *a priori* assumptions about the relative importance of specific site-wise amino acid differences and thus no weighting was used (e.g., I→N ≡ N→I) [15].

Initial analysis involved descriptive statistics of the data and correlation analysis. We performed a phylogenetic analysis of the sequence data by constructing a bootstrapped maximum likelihood tree in RAxMl with 500 bootstraps to depict the distribution/representation of the different topotypes in our data and to visualize relatedness of the serum/vaccine candidates cross-reacted in VNT assays to generate r_1_ values. Additionally, we performed a Spearman’s correlation analysis between p-amino acid distance and r1 scores. Following standard practice recommended by the World Organization for Animal Health (WOAH), we dichotomized r1 values at a threshold of 0.3, above which cross-neutralization is considered sufficient for vaccine matching [44].

## Training and testing machine learning models

Using a stepwise approach, we developed a random forest classification model with sub-sampling steps and tenfold cross validation to achieve the best model performance. Upon dichotomization of the r_1_ values into binomial data, there was an imbalance in the 0 (non-cross-reacting) vs 1 (cross-reacting) classes with more than twice as many observations in the 0 category as those in the 1 category (0 = 87 observations, 1 = 21 observations). We used the Synthetic Minority Oversampling Technique (SMOTE), which produced synthetic samples for the minority class to balance the distribution between cases [1] and non-cases (0), to lessen the impact of class imbalance in our training dataset. By using this technique, model bias toward the majority class was avoided, and minority instance recognition was enhanced. Nevertheless, we assessed the model using cross-validation and a different test dataset to reduce overfitting because SMOTE introduces synthetic data and might not adequately capture variation in the actual world, as explained below. Even though SMOTE increased sensitivity, in order to ensure that the model is generalizable outside of the training set, its effect on false positives was examined. Feature-reduction was then implemented to reduce the number of model features (i.e., predictors) using the Boruta algorithm to optimize model fitting. Upon concatenating all parts of the data into a single data frame, the final data frame had 216 columns (pairwise amino acid distance and 214 site-wise amino acid differences and potential sites for N-glycosylation). With highly dimensional data, machine learning models may struggle to achieve good model accuracy and prediction due to noise introduced as the model tries to optimize the estimated contribution of each model feature to the variation in the outcome (r_1_ class in our study). As such, the Boruta algorithm has been proposed as a way of eliminating correlated and redundant features from the model, thus reducing the number of model features needed in the final model and optimizing model performance. After parsing the data through Boruta, we included all model features classified as important or tentatively important, resulting in a data frame with 35 model features. These features were VP1, pairwise distances between amino acid sequences, and site-wise differences in amino acid positions [4,13,24,32,33,43,48,49,57,69, 97, 100, 124, 135, 139–141, 143, 145, 149–151, 154, 156, 159, 166, 173, 175, 195, 198, 199, 210, 213, 214] and potential N-glycosylation profile.

Subsequently we conducted a sequential data partitioning of the total 108 observations to effectively train and test our model. Initially, we split the dataset into two primary groups: the first consisting of 87 observations (approximately 80% of the total) and the second consisting of 21 observations (the remaining 20%). This initial division was designed to segregate the bulk of the data for extensive training and preliminary testing, while setting aside a smaller subset for final validation. We then further segmented the 87 observations, allocating 70 observations (about 80% of the 87) to form the training set and the remaining 17 observations to create the first test set. This structured approach allowed us to train the model while ensuring robust testing through two distinct test sets, thereby enhancing our evaluation of the model’s performance across different datasets. Using *caret* [57] and *randomForest* [58] packages in R for model building, training and evaluation, including hyper-parameter tuning and 10-fold cross-validation, we trained the model on 500 individual learners (iterations), tested it, and made predictions as summarized in the results section. We based model performance on accuracy (overall percent of observations correctly classified as cross-reacting/non-cross-reacting $Acc.=\frac{\left(True\;positve)+(True\;negative\right)}{N\;(total\;obs.)}$), sensitivity (percent of high observations correctly classified - $Se.=\frac{True\;positive}{\left(True\;positive)+\;(False\;negative\right)}$), specificity (percent of low observations correctly classified - $Sp.=\frac{True\;negative}{\left(True\;positive)+\;(False\;positive\right)}$), positive and negative predictive values (proportion (%) of times the classification (non-cross-protecting-0 vs cross-protecting-1 - $PPV.=\frac{True\;positive}{\left(True\;negative)+\;(False\;positive\right)}$ & $NPV.=\frac{True\;negative}{\left(True\;negative)+\;(False\;negative\right)}$), and the F1 score (the harmonic mean of precision/PPV and recall/sensitivity $F1=2(\frac{PPV\;x\;Se}{PPV+\;Se})$).

Additionally, from the random forest model, model features were ranked in their importance to the performance of our model using mean decrease in model accuracy when a features’ data were randomized relative to the outcome (the relative prediction strength of a variable) and improvement in Gini index (measure of node impurity associated with a variable) when data were split on a variable [59,60]. This allowed us to highlight highly ranked amino acids in our VP1data, and considered the relative importance of different amino acid sites based on their role in improving model accuracy and node purity in outcome classification [15,61].

To test the performance of the machine learning algorithm’s predictions, we used an external set of sequences data from vaccine matching studies by Bankowska [18] in Pakistan, Eltahir et al., [19] in United Arab Emirates, Singanallur et al., [20] in Australia, and Tsefaye et al. [21] in Ethiopia. In total, the three studies provided 38 serum-virus pairs on which predictions were made and compared to published r_1_ values or observed cross-neutralization from the lab assays conducted by the researchers. Although these studies were done to investigate, among other things, the effectiveness of vaccines, they were selected because they met the same inclusion criteria used for training data—specifically, r1 values derived from WOAH-standardized VNT protocols and available VP1 sequences. While the external datasets are smaller in size, they represent a geographically diverse set of studies conducted independently from those used in model development, making them suitable for assessing generalizability. These data are part of the supplemental material provided (S1 Table).

## Supporting information

S1 Table Predicted cross-reaction classification between vaccine and field strains reported in vaccine matching experiments from studies conducted in the United Arab Emirates, Pakistan, Australia, and Ethiopia. Model predictions (based on r₁ ≥ 0.3 threshold) are compared with published laboratory results for each serum-virus pair.
(DOCX)

S2 Table Summary of accession numbers of virus and vaccine/serum isolates included in the training dataset. Viruses were obtained from four published studies (Mahapatra et al., 2017; Tesfaye et al., 2020; Upadhyaya et al., 2021; Yang et al., 2014), representing multiple topotypes and countries.
(DOCX)

S1 Fig Maximum likelihood tree depicting the phylogenetic distribution of 96 foot-and-mouth disease virus (FMDV) serotype O isolates. Reference serum strains (pink triangles), field strains (blue circles), and validation samples (gray squares) are shown. Colored bars on the right indicate topotype assignments. NK = Not Known.
(TIF)

S2 Fig Principal Component Analysis (PCA) plots comparing the original training set (left) and the SMOTE-augmented set (right). Green triangles represent cross-neutralizing pairs (r₁ ≥ 0.3), red circles represent non-cross-neutralizing pairs (r₁ < 0.3). Ellipses indicate 95% confidence regions. SMOTE expanded the feature space while preserving structure.
(TIF)

## References

[1] Zell R, Delwart E, Gorbalenya AE, Hovi T, King AMQ, Knowles NJ, et al. ICTV Virus Taxonomy Profile: Picornaviridae. J Gen Virol. 2017;98(10):2421–2. doi: 10.1099/jgv.0.000911 28884666 PMC5725991

[2] Knight-Jones TJD, McLaws M, Rushton J. Foot‐and‐mouth disease impact on smallholders ‐ what do we know, what don’t we know and how can we find out more?. Transbound Emerg Dis. 2017;64(4):1079. 27167976 10.1111/tbed.12507 PMC5516236

[3] He Y, Li K, Cao Y, Sun Z, Li P, Bao H, et al. Structures of Foot-and-mouth Disease Virus with neutralizing antibodies derived from recovered natural host reveal a mechanism for cross-serotype neutralization. PLoS Pathog. 2021;17(4):e1009507. doi: 10.1371/journal.ppat.1009507 33909694 PMC8081260

[4] Brito BP, Rodriguez LL, Hammond JM, Pinto J, Perez AM. Review of the Global Distribution of Foot-and-Mouth Disease Virus from 2007 to 2014. Transbound Emerg Dis. 2017;64(2):316–32. doi: 10.1111/tbed.12373 25996568

[5] Grubman MJ, Baxt B. Foot-and-mouth disease. Clin Microbiol Rev. 2004;17(2):465–93. doi: 10.1128/CMR.17.2.465-493.2004 15084510 PMC387408

[6] Ranaweera LT, Wijesundara UK, Jayarathne HS-M, Knowles N, Wadsworth J, Mioulet V, et al. Characterization of the FMDV-serotype-O isolates collected during 1962 and 1997 discloses new topotypes, CEY-1 and WCSA-1, and six new lineages. Sci Rep. 2019;9(1):14526. doi: 10.1038/s41598-019-51120-0 31601911 PMC6787213

[7] Brito BP, Perez AM, Capozzo AV. Accuracy of traditional and novel serology tests for predicting cross-protection in foot-and-mouth disease vaccinated cattle. Vaccine. 2014;32(4):433–6. doi: 10.1016/j.vaccine.2013.12.007 24342253

[8] OIE/FAO Foot-and-Mouth Disease Reference Laboratories Network. Foot-and-mouth disease European July-September 2022 quarterly report. 2022. http://www.wrlfmd.org

[9] Qiu J, Qiu T, Dong Q, Xu D, Wang X, Zhang Q, et al. Predicting the Antigenic Relationship of Foot-and-Mouth Disease Virus for Vaccine Selection Through a Computational Model. IEEE/ACM Trans Comput Biol Bioinform. 2021;18(2):677–85. doi: 10.1109/TCBB.2019.2923396 31217127

[10] Borley DW, Mahapatra M, Paton DJ, Esnouf RM, Stuart DI, Fry EE. Evaluation and use of in-silico structure-based epitope prediction with foot-and-mouth disease virus. PLoS One. 2013;8(5). 10.1371/journal.pone.0061122 PMC3646828 23667434

[11] Bari FD, Parida S, Asfor AS, Haydon DT, Reeve R, Paton DJ. Prediction and characterization of novel epitopes of serotype A foot-and-mouth disease viruses circulating in East Africa using site-directed mutagenesis. J Gen Virol. 2015;96(5):1033–41. 25614587 10.1099/vir.0.000051 PMC4631058

[12] Rahman T, Mahapatra M, Laing E, Jin Y. Evolutionary non-linear modelling for selecting vaccines against antigenically variable viruses. Bioinformatics. 2015;31(6):834–40. doi: 10.1093/bioinformatics/btu768 25414361

[13] Davies V, Reeve R, Harvey WT, Maree FF, Husmeier D. A sparse hierarchical Bayesian model for detecting relevant antigenic sites in virus evolution. Comput Stat. 2017;32(3):803–43.

[14] Reeve R, Blignaut B, Esterhuysen JJ, Opperman P, Matthews L, Fry EE, et al. Sequence-based prediction for vaccine strain selection and identification of antigenic variability in foot-and-mouth disease virus. PLoS Comput Biol. 2010;6(12):e1001027. doi: 10.1371/journal.pcbi.1001027 21151576 PMC3000348

[15] Zeller MA, Gauger PC, Arendsee ZW, Souza CK, Vincent AL, Anderson TK. Machine Learning Prediction and Experimental Validation of Antigenic Drift in H3 Influenza A Viruses in Swine. mSphere. 2021;6(2):e00920-20. doi: 10.1128/mSphere.00920-20 33731472 PMC8546707

[16] Bell SM, Katzelnick L, Bedford T. Dengue genetic divergence generates within-serotype antigenic variation, but serotypes dominate evolutionary dynamics. Elife. 2019. 10.7554/eLife.42496 PMC6731059 31385805

[17] Mansfield KL, Horton DL, Johnson N, Li L, Barrett ADT, Smith DJ, et al. Flavivirus-induced antibody cross-reactivity. J Gen Virol. 2011;92(Pt 12):2821–9. doi: 10.1099/vir.0.031641-0 21900425 PMC3352572

[18] Bachanek-Bankowska K, Wadsworth J, Henry E, Ludi AB, Bin-Tarif A, Statham B, et al. Genome Sequences of Antigenically Distinct Serotype O Foot-and-Mouth Disease Viruses from Pakistan. Microbiol Resour Announc. 2019;8(3):e01397-18. doi: 10.1128/MRA.01397-18 30687826 PMC6346158

[19] Eltahir YM, Ishag HZA, Parekh K, Wood BA, Ludi A, King DP, et al. Foot and Mouth Disease Vaccine Matching and Post-Vaccination Assessment in Abu Dhabi, United Arab Emirates. Vet Sci. 2024;11(6):272. doi: 10.3390/vetsci11060272 38922019 PMC11209342

[20] Singanallur NB, Dekker A, Eblé PL, van Hemert-Kluitenberg F, Weerdmeester K, Horsington JJ, et al. Emergency FMD Serotype O Vaccines Protect Cattle against Heterologous Challenge with a Variant Foot-and-Mouth Disease Virus from the O/ME-SA/Ind2001 Lineage. Vaccines (Basel). 2021;9(10):1110. doi: 10.3390/vaccines9101110 34696216 PMC8537456

[21] Tesfaye Y, Khan F, Gelaye E. Vaccine matching and antigenic variability of foot-and-mouth disease virus serotypes O and A from 2018 Ethiopian isolates. Int Microbiol. 2022;25(1):47–59. doi: 10.1007/s10123-021-00178-w 34224048

[22] Paton DJ, Reeve R, Capozzo AV, Ludi A. Estimating the protection afforded by foot-and-mouth disease vaccines in the laboratory. Vaccine. 2019;37(37):5515–24. doi: 10.1016/j.vaccine.2019.07.102 31405637

[23] Lavoria M ángeles, Di-Giacomo S, Bucafusco D, Franco-Mahecha OL, Pérez-Filgueira DM, Capozzo AV. Avidity and subtyping of specific antibodies applied to the indirect assessment of heterologous protection against Foot-and-Mouth Disease Virus in cattle. Vaccine. 2012;30(48):6845–50. 23000129 10.1016/j.vaccine.2012.09.011

[24] Robiolo B, La Torre J, Maradei E, Beascoechea CP, Perez A, Seki C, et al. Confidence in indirect assessment of foot-and-mouth disease vaccine potency and vaccine matching carried out by liquid phase ELISA and virus neutralization tests. Vaccine. 2010;28(38):6235–41. doi: 10.1016/j.vaccine.2010.07.012 20643090

[25] Mattion N, Goris N, Willems T, Robiolo B, Maradei E, Beascoechea CP, et al. Some guidelines for determining foot-and-mouth disease vaccine strain matching by serology. Vaccine. 2009;27(5):741–7. doi: 10.1016/j.vaccine.2008.11.026 19041355

[26] Neher RA, Russell CA, Shraiman BI. Predicting evolution from the shape of genealogical trees. Elife. 2014;3:e03568. doi: 10.7554/eLife.03568 25385532 PMC4227306

[27] Xia YL, Li W, Li Y, Ji XL, Fu YX, Liu SQ. A deep learning approach for predicting antigenic variation of influenza A H3N2. Comput Math Methods Med. 2021;2021:1–10. 10.1155/2021/9997669 PMC8541863 34697557

[28] Forghani M, Khachay M. Convolutional Neural Network Based Approach to in Silico Non-Anticipating Prediction of Antigenic Distance for Influenza Virus. Viruses. 2020;12(9):1019. doi: 10.3390/v12091019 32932748 PMC7551508

[29] Lee EK, Tian H, Nakaya HI. Antigenicity prediction and vaccine recommendation of human influenza virus A (H3N2) using convolutional neural networks. Hum Vaccin Immunother. 2020;16(11):2690–708. doi: 10.1080/21645515.2020.1734397 32750260 PMC7734114

[30] Makau DN, Lycett S, Michalska-Smith M, Paploski IAD, Cheeran MCJ, Craft ME, et al. Ecological and evolutionary dynamics of multi-strain RNA viruses. Nature Ecology & Evolution. 2022;:1–9. 36138206 10.1038/s41559-022-01860-6

[31] King SM, Bryan SP, Hilchey SP, Wang J, Zand MS. First Impressions Matter: Immune Imprinting and Antibody Cross-Reactivity in Influenza and SARS-CoV-2. Pathogens. 2023;12(2):169. doi: 10.3390/pathogens12020169 36839441 PMC9967769

[32] Warter L, Appanna R, Fink K. Human poly- and cross-reactive anti-viral antibodies and their impact on protection and pathology. Immunol Res. 2012;53(1–3):148–61. doi: 10.1007/s12026-012-8268-8 22434513

[33] Huang YJS, Higgs S, Vanlandingham DL. Arbovirus-mosquito vector-host interactions and the impact on transmission and disease pathogenesis of arboviruses. Front Microbiol. 2019;10(JAN):434047. 10.3389/fmicb.2019.00022 PMC6351451 30728812

[34] Islam MR, Rahman MS, Amin MAl, Alam ASMRU, Siddique MA, Sultana M, et al. Evidence of combined effect of amino acid substitutions within G-H and B-C loops of VP1 conferring serological heterogeneity in foot-and-mouth disease virus serotype A. Transbound Emerg Dis. 2021;68(2):375–84. 32543041 10.1111/tbed.13687

[35] Jumper J, Evans R, Pritzel A, Green T, Figurnov M, Ronneberger O, et al. Highly accurate protein structure prediction with AlphaFold. Nature. 2021;596(7873):583–9. doi: 10.1038/s41586-021-03819-2 34265844 PMC8371605

[36] Berman HM, Westbrook J, Feng Z, Gilliland G, Bhat TN, Weissig H, et al. The Protein Data Bank. Nucleic Acids Res. 2000;28(1):235–42. doi: 10.1093/nar/28.1.235 10592235 PMC102472

[37] Logan D, Abu-Ghazaleh R, Blakemore W, Curry S, Jackson T, King A. Structure of a major immunogenic site on foot-and-mouth disease virus. Nature. 1993;362(6420):566–8. 8385272 10.1038/362566a0

[38] Cardoso N, Eschbaumer M, Capozzo AV. An IgG1 single-dilution avidity ELISA predicts cross-protection against heterologous foot-and-mouth disease virus challenge after vaccination. Vaccine. 2024;42(25):126066. doi: 10.1016/j.vaccine.2024.06.033 38876835

[39] Maree FF, Blignaut B, Esterhuysen JJ, de Beer TAP, Theron J, O’Neill HG, et al. Predicting antigenic sites on the foot-and-mouth disease virus capsid of the South African Territories types using virus neutralization data. J Gen Virol. 2011;92(Pt 10):2297–309. doi: 10.1099/vir.0.032839-0 21697350

[40] Tesfaye Y, Khan F, Yami M, Wadsworth J, Knowles NJ, King DP, et al. A vaccine-matching assessment of different genetic variants of serotype O foot-and-mouth disease virus isolated in Ethiopia between 2011 and 2014. Arch Virol. 2020;165(8):1749–57. doi: 10.1007/s00705-020-04662-y 32435857

[41] Upadhyaya S, Mahapatra M, Mioulet V, Parida S. Molecular basis of antigenic drift in serotype O foot-and-mouth disease viruses (2013-2018) from Southeast Asia. Viruses. 2021;13(9). 10.3390/v13091886 PMC8473337 34578467

[42] Yang M, Xu W, Goolia M, Zhang Z. Characterization of monoclonal antibodies against foot-and-mouth disease virus serotype O and application in identification of antigenic variation in relation to vaccine strain selection. Virol J. 2014;11:136. doi: 10.1186/1743-422X-11-136 25085313 PMC4125342

[43] Gubbins S, Paton DJ, Dekker A, Ludi AB, Wilsden G, Browning CFJ, et al. Predicting cross-protection against foot-and-mouth disease virus strains by serology after vaccination. Front Vet Sci. 2022. 10.3389/fvets.2022.1027006 PMC9751447 36532344

[44] World Organization for Animal Health. Foot and mouth disease (infection with foot and mouth disease virus). OIE Terrestrial Manual. 2022. 1–34.

[45] Larsson A. AliView: a fast and lightweight alignment viewer and editor for large datasets. Bioinformatics. 2014;30(22):3276–8. doi: 10.1093/bioinformatics/btu531 25095880 PMC4221126

[46] Kumar S, Stecher G, Li M, Knyaz C, Tamura K. Molecular Biology and Evolution. 2018;35(6):1547–9. 29722887 10.1093/molbev/msy096 PMC5967553

[47] Zuckerkandl E, Pauling L. Evolutionary divergence and convergence in proteins. Evolving genes and proteins. Elsevier. 1965. 97–166.

[48] CRAN - Package stringr. https://stringr.tidyverse.org/authors.html. 2022 June 17.

[49] Keck F. Handling biological sequences in R with the bioseq package. Methods Ecol Evol. 2020;11(12):1728–32. doi: 10.1111/2041-210x.13490

[50] Paradis E, Schliep K. Ape 5.0: an environment for modern phylogenetics and evolutionary analyses in R. Bioinformatics. 2019;35(3):526–8. 30016406 10.1093/bioinformatics/bty633

[51] CRAN - Package tidysq. https://cran.rstudio.com/web/packages/tidysq/index.html. 2022 June 17.

[52] R Core Team. R: A Language and Environment for Statistical Computing. https://www.r-project.org/. 2022.

[53] Chirkova ZV, Filimonov SI, Prituzhalov IV, Vasanov EA, Abramov IG. Synthesis of chalcones from 3-formyl-substituted pyrrolo[3,4-f]indole-5,7-diones. Russ Chem Bull. 2017;66(5):882–5.

[54] Paploski IAD, Makau DN, Pamornchainavakul N, Baker JP, Schroeder D, Rovira A, et al. Potential Novel N-Glycosylation Patterns Associated with the Emergence of New Genetic Variants of PRRSV-2 in the U.S. Vaccines (Basel). 2022;10(12):2021. doi: 10.3390/vaccines10122021 36560431 PMC9787953

[55] Yao Y, Li X, Liao B, Huang L, He P, Wang F, et al. Predicting influenza antigenicity from Hemagglutintin sequence data based on a joint random forest method. Sci Rep. 2017;7(1):1545. doi: 10.1038/s41598-017-01699-z 28484283 PMC5431489

[56] Bedford T, Suchard MA, Lemey P, Dudas G, Gregory V, Hay AJ. Integrating influenza antigenic dynamics with molecular evolution. Elife. 2014;3. 10.7554/eLife.01914 PMC3909918 24497547

[57] Kuhn M. Building predictive models in R using the caret package. J Stat Softw. 2008;28(5):1–26.27774042

[58] Liaw A, Wiener M. Classification and Regression by randomForest. R News. 2002;2(3):18–22.

[59] Hastie T, Tibshirani R, Friedman J. Random Forests. Springer Series in Statistics. Springer New York. 2008. 587–604. doi: 10.1007/978-0-387-84858-7_15

[60] Machado G, Mendoza MR, Corbellini LG. What variables are important in predicting bovine viral diarrhea virus? A random forest approach. Vet Res. 2015;46(1):85. doi: 10.1186/s13567-015-0219-7 26208851 PMC4513962

[61] Makau DN, Prieto C, Martínez-Lobo FJ, Paploski IAD, VanderWaal K. Predicting antigenic distance from genetic data for PRRSV-Type 1: applications of machine learning. Microbiol Spectr. 2023;11(1). 10.1128/spectrum.04085-22 PMC9927307 36511691

