# Supplementary material for: Opportunities for machine learning to predict cross-neutralization in FMDV serotype O
Source: PLoS Comput Biol. 2025 Sep 17;21(9):e1013491. doi: 10.1371/journal.pcbi.1013491 (PMC12456779; doi:10.1371/journal.pcbi.1013491)
Supplement: S1 Table — Model predictions (based on r₁ ≥ 0.3 threshold) are compared with published laboratory results for each serum-virus pair. (DOCX) [file pcbi.1013491.s001.docx]

S1 Table: Predicted cross-reaction classification between vaccine and field strains reported in vaccine matching experiments by studies in United Arab Emirates (1), Pakistan (2), Australia (3), and Ethiopia (4) respectively.

| **Field strain** | **Vaccine strain** | **Model r1 prediction** | **Published Lab results** |
| --- | --- | --- | --- |
| **Eltahir et al. study** | | | |
| OR425051.1_UAE/1/2021 | JF968170.1_3039 | >0.3 | >0.3 |
| OR425057.1_UAE/15/2021 | JF968170.1_3039 | <0.3 | >0.3 |
| OR425053.1_UAE/9/2021 | JF968170.1_3039 | >0.3 | >0.3 |
| OR425051.1_UAE/1/2021 | LQ465469.1_Campos | >0.3 | >0.3 |
| OR425057.1_UAE/15/2021 | LQ465469.1_Campos | >0.3 | >0.3 |
| OR425053.1_UAE/9/2021 | LQ465469.1_Campos | >0.3 | >0.3 |
| OR425051.1_UAE/1/2021 | AY593823.1_O1_Manisa | >0.3 | >0.3 |
| OR425057.1_UAE/15/2021 | AY593823.1_O1_Manisa | >0.3 | >0.3 |
| OR425053.1_UAE/9/2021 | AY593823.1_O1_Manisa | >0.3 | >0.3 |
| OR425051.1_UAE/1/2021 | KF321732.1_PanAsia-2 | >0.3 | >0.3 |
| OR425057.1_UAE/15/2021 | KF321732.1_PanAsia-2 | >0.3 | >0.3 |
| OR425053.1_UAE/9/2021 | KF321732.1_PanAsia-2 | >0.3 | >0.3 |
| OR425051.1_UAE/1/2021 | MT443823.1_TUR/5/2009 | >0.3 | >0.3 |
| OR425057.1_UAE/15/2021 | MT443823.1_TUR/5/2009 | >0.3 | >0.3 |
| OR425053.1_UAE/9/2021 | MT443823.1_TUR/5/2009 | >0.3 | >0.3 |
| **Bachanek-Bankowska study** | | | |
| MH784405.1_PAK/14/2017 | JF968170.1_3039 | >0.3 | >0.3 |
| MH784405.1_PAK/14/2017 | AY593823.1_o_1manisa | >0.3 | >0.3 |
| MH784405.1_PAK/14/2017 | MT443823.1_TUR/5/2009 | >0.3 | >0.3 |
| MH784403.1_PAK/10/2016 | JF968170.1_3039 | >0.3 | <0.3 |
| MH784403.1_PAK/10/2016 | AY593823.1_o_1manisa | >0.3 | <0.3 |
| MH784403.1_PAK/10/2016 | MT443823.1_TUR/5/2009 | >0.3 | <0.3 |
| MH784404.1_PAK/4/2017 | JF968170.1_3039 | >0.3 | <0.3 |
| MH784404.1_PAK/4/2017 | AY593823.1_o_1manisa | >0.3 | <0.3 |
| MH784404.1_PAK/4/2017 | MT443823.1_TUR/5/2009 | >0.3 | <0.3 |
| **Singanallur et al. study** | | | |
| KY696708.1_O/ME-SA/Ind-2001d | JF968170.1_3039 | >0.3 | >0.3 |
| KY696708.1_O/ME-SA/Ind-2001d | AY593823.1_o_1manisa | >0.3 | <0.3 |
| **Tsefaye et al. study** | | | |
| MN987470.1_ETH/21/2018 | FJ798108.1_ETH/38/2005 | >0.3 | >0.3 |
| MN987469.1_ETH/20/2018 | FJ798108.1_ETH/38/2005 | >0.3 | >0.3 |
| MN987474.1_ETH/25/2018 | FJ798108.1_ETH/38/2005 | >0.3 | >0.3 |
| MN987471.1_ETH/22/2018 | FJ798108.1_ETH/38/2005 | >0.3 | >0.3 |
| MN987468.1_ETH/19/2018 | FJ798108.1_ETH/38/2005 | >0.3 | >0.3 |

References

1. Eltahir YM, Ishag HZA, Parekh K, Wood BA, Ludi A, King DP, et al. Foot and Mouth Disease Vaccine Matching and Post-Vaccination Assessment in Abu Dhabi, United Arab Emirates. Vet Sci 2024, Vol 11, Page 272 [Internet]. 2024 Jun 14 [cited 2024 Sep 24];11(6):272. Available from: https://www.mdpi.com/2306-7381/11/6/272/htm

2. Bachanek-Bankowska K, Wadsworth J, Henry E, Ludi AB, Bin-Tarif A, Statham B, et al. Genome Sequences of Antigenically Distinct Serotype O Foot-and-Mouth Disease Viruses from Pakistan. Bruno V, editor. Microbiol Resour Announc [Internet]. 2019 Jan 17 [cited 2023 Nov 14];8(3). Available from: https://journals.asm.org/doi/10.1128/mra.01397-18

3. Singanallur NB, Dekker A, Eblé PL, van Hemert-Kluitenberg F, Weerdmeester K, Horsington JJ, et al. Emergency FMD Serotype O Vaccines Protect Cattle against Heterologous Challenge with a Variant Foot-and-Mouth Disease Virus from the O/ME-SA/Ind2001 Lineage. Vaccines [Internet]. 2021 Sep 29 [cited 2024 Sep 24];9(10):1110. Available from: https://www.mdpi.com/2076-393X/9/10/1110/htm

4. Tesfaye Y, Khan F, Gelaye E. Vaccine matching and antigenic variability of foot-and-mouth disease virus serotypes O and A from 2018 Ethiopian isolates. Int Microbiol [Internet]. 2022 Jan 5 [cited 2024 Sep 24];25(1):47–59. Available from: https://link.springer.com/10.1007/s10123-021-00178-w

1. Eltahir YM, Ishag HZA, Parekh K, Wood BA, Ludi A, King DP, et al. Foot and Mouth Disease Vaccine Matching and Post-Vaccination Assessment in Abu Dhabi, United Arab Emirates. Vet Sci 2024, Vol 11, Page 272 [Internet]. 2024 Jun 14 [cited 2024 Sep 24];11(6):272. Available from: https://www.mdpi.com/2306-7381/11/6/272/htm

2. Bachanek-Bankowska K, Wadsworth J, Henry E, Ludi AB, Bin-Tarif A, Statham B, et al. Genome Sequences of Antigenically Distinct Serotype O Foot-and-Mouth Disease Viruses from Pakistan. Bruno V, editor. Microbiol Resour Announc [Internet]. 2019 Jan 17 [cited 2023 Nov 14];8(3). Available from: https://journals.asm.org/doi/10.1128/mra.01397-18

3. Singanallur NB, Dekker A, Eblé PL, van Hemert-Kluitenberg F, Weerdmeester K, Horsington JJ, et al. Emergency FMD Serotype O Vaccines Protect Cattle against Heterologous Challenge with a Variant Foot-and-Mouth Disease Virus from the O/ME-SA/Ind2001 Lineage. Vaccines [Internet]. 2021 Sep 29 [cited 2024 Sep 24];9(10):1110. Available from: https://www.mdpi.com/2076-393X/9/10/1110/htm

4. Tesfaye Y, Khan F, Gelaye E. Vaccine matching and antigenic variability of foot-and-mouth disease virus serotypes O and A from 2018 Ethiopian isolates. Int Microbiol [Internet]. 2022 Jan 5 [cited 2024 Sep 24];25(1):47–59. Available from: https://link.springer.com/10.1007/s10123-021-00178-w
