# Supplementary material for: Opportunities for machine learning to predict cross-neutralization in FMDV serotype O
Source: PLoS Comput Biol. 2025 Sep 17;21(9):e1013491. doi: 10.1371/journal.pcbi.1013491 (PMC12456779; doi:10.1371/journal.pcbi.1013491)
Supplement: S2 Table — Viruses were obtained from four published studies (Mahapatra et al., 2017; Tesfaye et al., 2020; Upadhyaya et al., 2021; Yang et al., 2014), representing multiple topotypes and countries. (DOCX) [file pcbi.1013491.s002.docx]

S2 Table: Summary of accession numbers of virus and vaccine/serum isolates included in this study. All viruses were obtained from studies by these 4 studies(1–4)

| Study (reference) | Year | # of r₁ pairs | Topotypes represented | Accession Numbers |
| --- | --- | --- | --- | --- |
| Mahapatra M et al. Vaccine (2017) | 2011 | 4 | SAT 1, SAT 2, SAT 3 | KJ831676.1, DQ164880.1, DQ164881.1, KM243162.1, KM243163.1 |
| Tesfaye Y et al. Arch Virol (2020) | 2020 | 39 | EA-3, EA-4 | MZ851288.1, MZ851289.1, MZ851290.1, MZ851291.1, MZ851292.1, MZ851293.1, MZ851294.1, MZ851295.1, MZ851296.1, MZ851297.1, MZ851298.1, MZ851299.1, MZ851300.1, MZ851301.1, MZ851302.1, MZ851303.1 |
| Yang M et al. Virology J (2014) | 2014 | 30 | ME-SA, EA-3, SEA, WCSA | KJ606977.1, KJ606980.1, KJ606981.1, KJ606983.1, KJ606984.1, KJ606978.1, KC519630.1, KJ606979.1, KJ606982.1 |
| Upadhyaya S et al. Viruses (2021) | 2021 | 35 | PanAsia-2, IND-R2/75 | MN518164.1, MN518166.1, MN518152.1, MN518153.1, MN518154.1, MN518157.1, MN518149.1, MN518151.1, MN518142.1, MN518146.1, MN518147.1, MN518143.1, MN518155.1, MN518144.1, MN518145.1, MN518148.1 |

List of serum/vaccines used in the model**:** KF321732.1, KJ831676.1, AF204276.1, AY593823.1, FJ798108.1, KR401172.1

References

1. Mahapatra M, Upadhyaya S, Aviso S, Babu A, Hutchings G, Parida S. Selection of vaccine strains for serotype O foot-and-mouth disease viruses (2007-2012) circulating in Southeast Asia, East Asia and Far East. Vaccine [Internet]. 2017 Dec 18 [cited 2024 Sep 17];35(51):7147–53. Available from: http://www.ncbi.nlm.nih.gov/pubmed/29157957

2. Tesfaye Y, Khan F, Yami M, Wadsworth J, Knowles NJ, King DP, et al. A vaccine-matching assessment of different genetic variants of serotype O foot-and-mouth disease virus isolated in Ethiopia between 2011 and 2014. Arch Virol [Internet]. 2020 Aug 1 [cited 2023 Nov 14];165(8):1749–57. Available from: https://pubmed.ncbi.nlm.nih.gov/32435857/

3. Upadhyaya S, Mahapatra M, Mioulet V, Parida S. Molecular Basis of Antigenic Drift in Serotype O Foot-and-Mouth Disease Viruses (2013-2018) from Southeast Asia. Viruses [Internet]. 2021 Sep 1 [cited 2023 Nov 14];13(9). Available from: https://pubmed.ncbi.nlm.nih.gov/34578467/

4. Yang M, Xu W, Goolia M, Zhang Z. Characterization of monoclonal antibodies against foot-and-mouth disease virus serotype O and application in identification of antigenic variation in relation to vaccine strain selection. Virol J [Internet]. 2014 Aug 1 [cited 2023 Nov 14];11(1):136. Available from: /pmc/articles/PMC4125342/
