# Supplementary figures and images for: Opportunities for machine learning to predict cross-neutralization in FMDV serotype O
Source: PLoS Comput Biol. 2025 Sep 17;21(9):e1013491. doi: 10.1371/journal.pcbi.1013491 (PMC12456779; doi:10.1371/journal.pcbi.1013491)

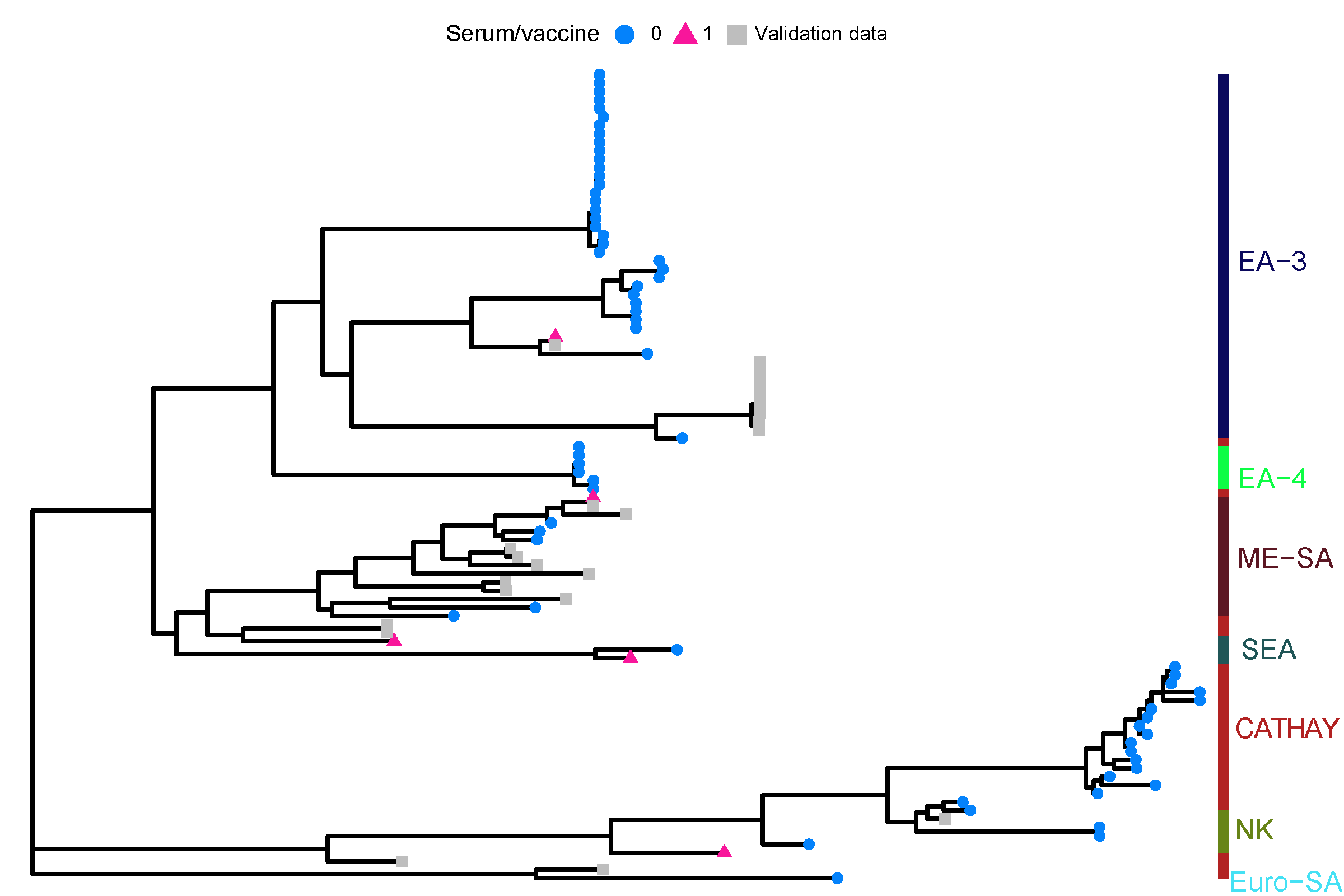

Supplement: S1 Fig — Reference serum strains (pink triangles), field strains (blue circles), and validation samples (gray squares) are shown. Colored bars on the right indicate topotype assignments. NK = Not Known. (TIF) [file pcbi.1013491.s003.tif]

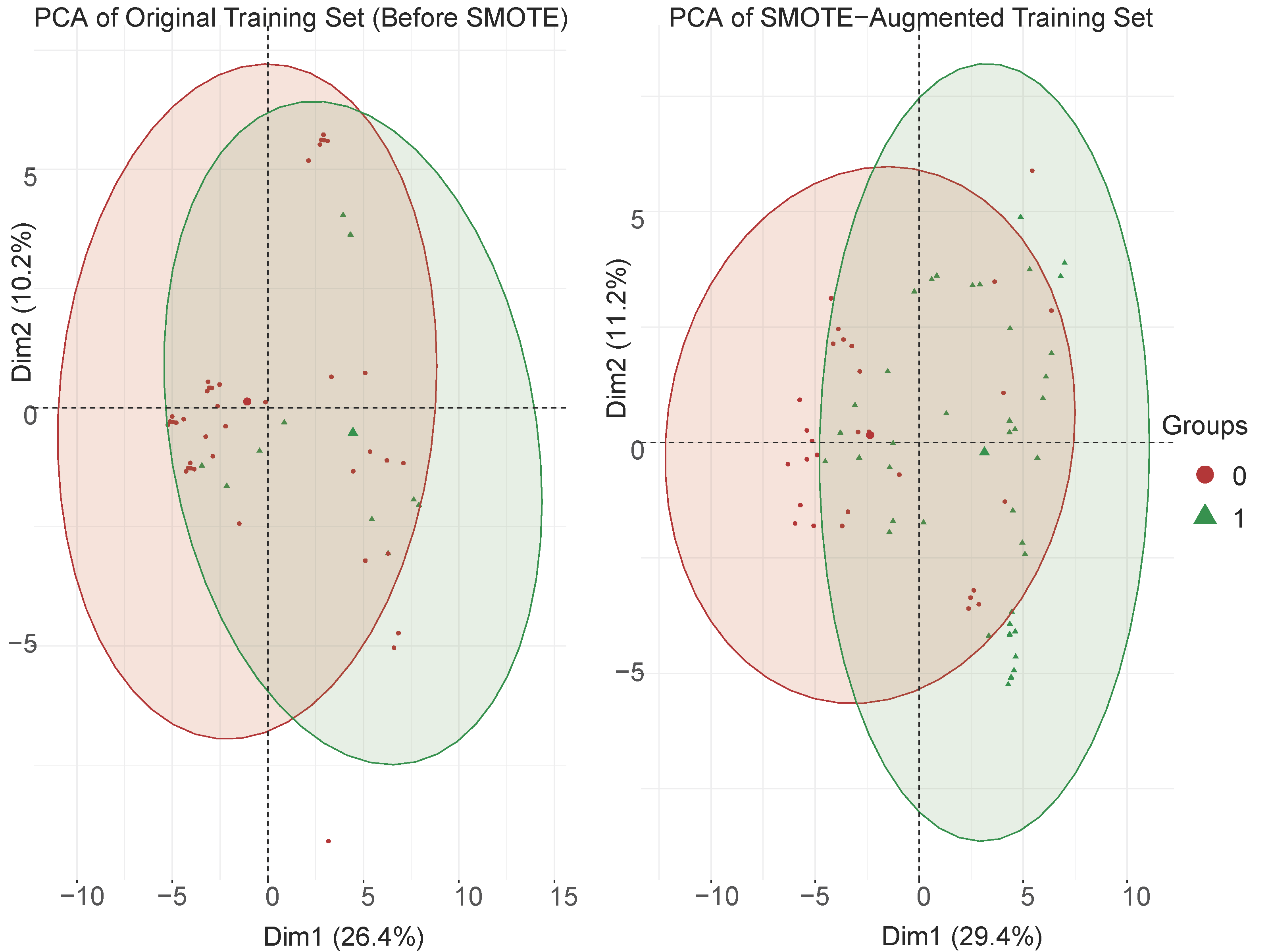

Supplement: S2 Fig — Green triangles represent cross-neutralizing pairs (r₁ ≥ 0.3), red circles represent non-cross-neutralizing pairs (r₁ < 0.3). Ellipses indicate 95% confidence regions. SMOTE expanded the feature space while preserving structure. (TIF) [file pcbi.1013491.s004.tif]
